# Supplementary material for: Expression and Kinetics of Endogenous Cannabinoids in the Brain and Spinal Cord of a Spare Nerve Injury (SNI) Model of Neuropathic Pain
Source: Cells. 2022 Dec 19;11(24):4130. doi: 10.3390/cells11244130 (PMC9776457; doi:10.3390/cells11244130)
Supplement: Supplementary file 1 [file cells-11-04130-s001.zip › cells-2027134-supplementary.pdf]

Figure S1

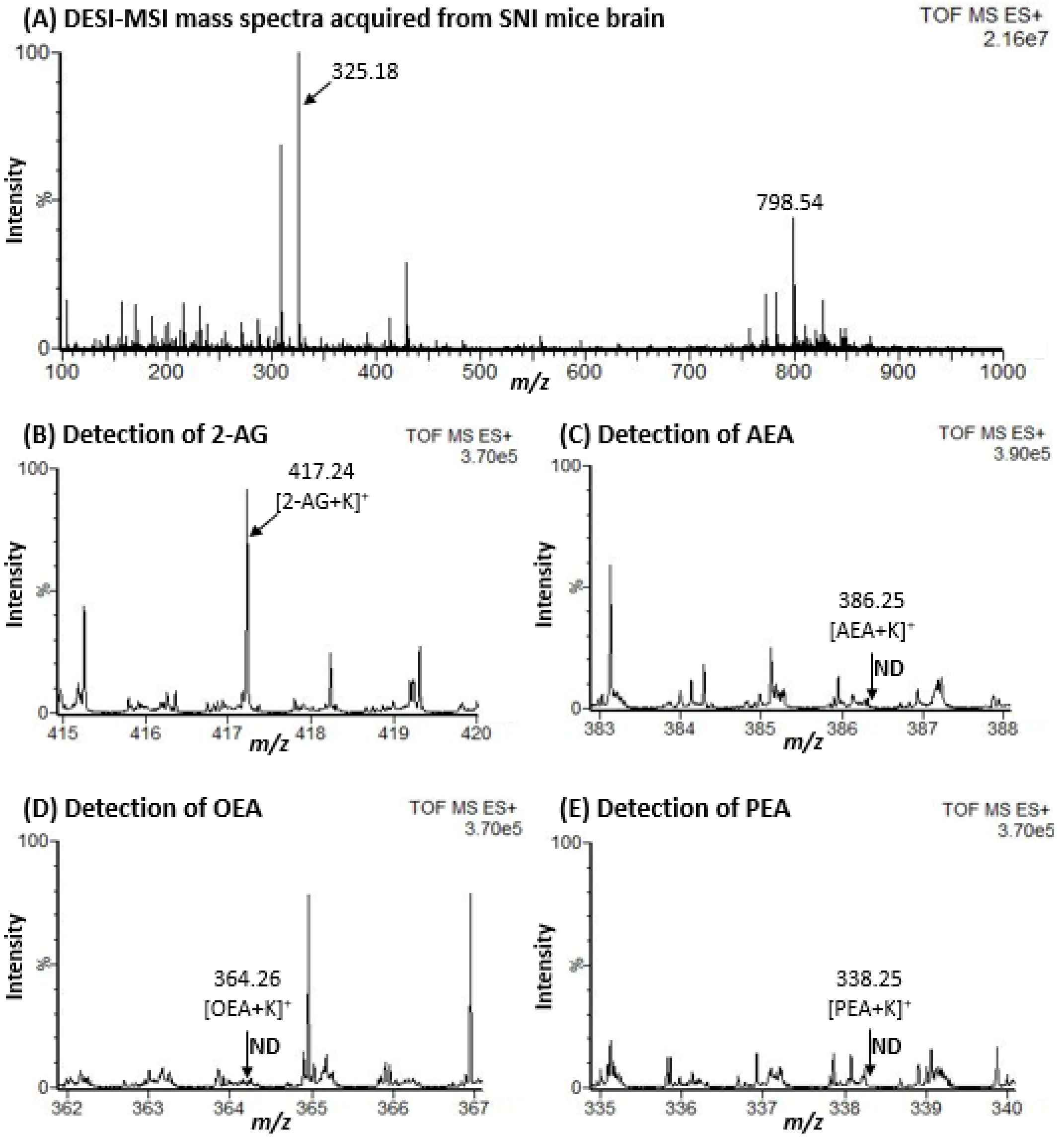

Figure S1: DESI-MSI spectra acquired from the coronal section of SNI mice brain. (A) Whole DESI-MSI spectra acquired from SNI mice brain. (B) m/z peak corresponding to 2-arachidonoylglycerol (2-AG) was detected in DESI-MSI spectra. (C-E) m/z peaks corresponding to anandamide (AEA), oleoylethanolamide (OEA), and palmitoylethanolamide (PEA) were not detected (ND) in the DESI-MSI spectra.
